# Understanding the structure and dynamics of hydrogenases by ultrafast and two-dimensional infrared spectroscopy†
†Electronic supplementary information (ESI) available: Supplementary figures. Description of the Morse fit. Analysis of CN stretch vibrational relaxation. References. See DOI: 10.1039/c9sc02851j


**DOI:** 10.1039/c9sc02851j

**Published:** 2019-08-05

**Authors:** Marius Horch, Janna Schoknecht, Solomon L. D. Wrathall, Gregory M. Greetham, Oliver Lenz, Neil T. Hunt

**Affiliations:** a Department of Chemistry , York Biomedical Research Institute , University of York , Heslington , York , YO10 5DD , UK . Email: marius.horch@york.ac.uk; b Institut für Chemie , Technische Universität Berlin , Straße des 17. Juni 135 , Berlin , D-10623 , Germany; c STFC Central Laser Facility, Research Complex at Harwell , Rutherford Appleton Laboratory , Harwell Science and Innovation Campus , Didcot , Oxford , OX110PE , UK

## Abstract

Hydrogenases are valuable model enzymes for sustainable energy conversion approaches using H_2_, but rational utilization of these base-metal biocatalysts requires a detailed understanding of the structure and dynamics of their complex active sites. The intrinsic CO and CN^–^ ligands of these metalloenzymes represent ideal chromophores for infrared (IR) spectroscopy, but structural and dynamic insight from conventional IR absorption experiments is limited. Here, we apply ultrafast and two-dimensional (2D) IR spectroscopic techniques, for the first time, to study hydrogenases in detail. Using an O_2_-tolerant [NiFe] hydrogenase as a model system, we demonstrate that IR pump–probe spectroscopy can explore catalytically relevant ligand bonding by accessing high-lying vibrational states. This ultrafast technique also shows that the protein matrix is influential in vibrational relaxation, which may be relevant for energy dissipation from the active site during fast reaction steps. Further insights into the relevance of the active site environment are provided by 2D-IR spectroscopy, which reveals equilibrium dynamics and structural constraints imposed on the H_2_-accepting intermediate of [NiFe] hydrogenases. Both techniques offer new strategies for uniquely identifying redox-structural states in complex catalytic mixtures *via* vibrational quantum beats and 2D-IR off-diagonal peaks. Together, these findings considerably expand the scope of IR spectroscopy in hydrogenase research, and new perspectives for the characterization of these enzymes and other (bio-)organometallic targets are presented.

## Abstract 2

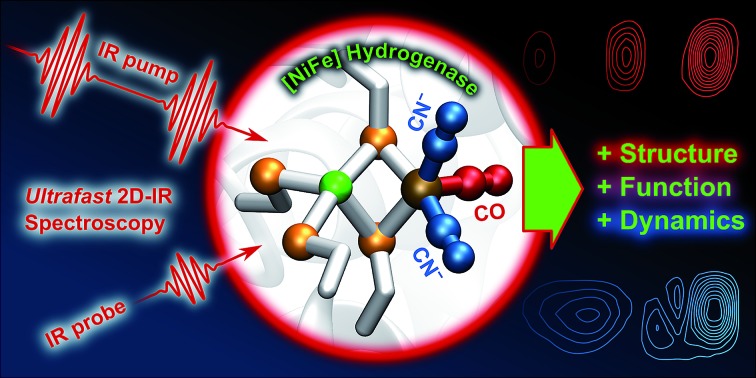
A proof-of-concept study on a catalytic [NiFe] intermediate reveals structural and dynamical details of hydrogenases by ultrafast and two-dimensional infrared spectroscopies.

## Introduction

Dihydrogen (H_2_) is a clean fuel that releases large amounts of free energy but no greenhouse gases upon combustion. Catalysing the reversible cleavage of H_2_, hydrogenases are valuable model enzymes for sustainable energy conversion approaches.^1^ Their utilization as biotechnological targets or blueprints for bio-inspired chemistry, however, requires a thorough understanding of the structural, dynamic, and mechanistic traits of these metalloenzymes. Several central aspects of hydrogenase function are still far from understood, and, thus, there is a demand for new spectroscopic techniques that can reveal ever greater levels of molecular detail.

**Fig. 1:**
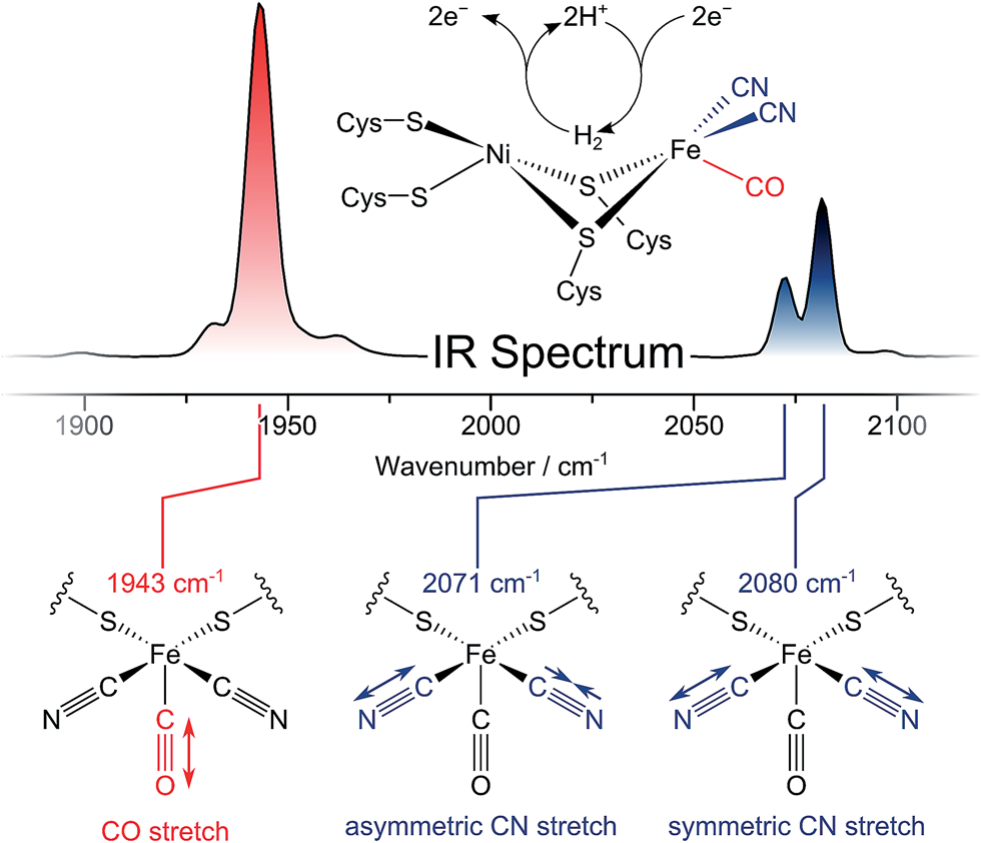
(Top) IR absorption spectrum and active site structure of oxidized *Re*RH in the Ni_a_-S state, the H_2_-accepting intermediate of [NiFe] hydrogenases. (Bottom) Visualization of CO (red) and CN (blue) stretching modes detected in the IR spectra of [NiFe] hydrogenases. Vibrational frequencies of these modes are sensitive to details of the active site, so that each redox-structural [NiFe] state exhibits a distinct signature of three absorption signals. Listed Frequencies refer to the Ni_a_-S state of *Re*RH.

Infrared (IR) spectroscopy has played a central role in the characterization of hydrogenases since the first detection of biologically unprecedented CO and CN^–^ ligands in the active sites of these enzymes (Fig. 1, top).^2^–^5^ Both types of ligands exhibit structurally sensitive bond stretching vibrations in an otherwise featureless region of the IR spectrum (Fig. 1, top).^3^,^6^ Thus, IR spectroscopy has been widely used to monitor transitions between redox-structural states of hydrogenase active sites under various experimental conditions.^7^,^8^ So far, however, all IR studies on these enzymes have focused on linear absorption experiments and the interpretation of vibrational frequencies. Due to the strict localization of CO/CN stretch modes and the multitude of determinants that govern their vibrational frequencies, structural information from this single set of observables is inherently limited. Moreover, time-resolved insights into hydrogenases are still scarce. While non-equilibrium studies have provided information on active site state interconversions,^9^ they fail to provide dynamic insights into the states themselves, as would be obtained under equilibrium or steady state conditions. Thus, *individual* redox-structural states have only been characterised in a time-averaged manner so far.

Here, we apply ultrafast pump–probe and two-dimensional (2D) IR techniques, for the first time, to study the active site of a hydrogenase. Using ultrashort laser pulses and multiple light–matter interactions, these non-linear experiments provide access to several unexplored observables that enhance our understanding of the structure and dynamics of hydrogenases under biologically relevant conditions.^10^–^12^ To illustrate the merit of ultrafast IR techniques, we utilize the regulatory [NiFe] hydrogenase from *Ralstonia eutropha* (*Re*RH), which proved to be a valuable model system.^13^–^15^ Like other [NiFe] hydrogenases,^7^ this enzyme harbours a heterobimetallic active site containing two metal ions, Ni and Fe, that are coordinated by four cysteine thiolates (Fig. 1, top).^13^,^14^,^16^–^18^ In addition, the low-spin Fe^II^ ion (S = 0)^14^ carries one CO and two CN^–^ ligands, as observed for all known [NiFe] hydrogenases.^7^,^19^–^23^ Being an H_2_-sensing hydrogenase, *Re*RH exhibits comparably low catalytic activity for both H_2_ activation and evolution,^17^ most likely due to unusual properties of its FeS clusters.^14^ On the other hand, *Re*RH is not inhibited by dioxygen (O_2_),^16^,^17^,^24^ and only few (virtually pure) catalytically active species are observed under most relevant conditions.^13^,^16^–^18^ Specifically, the as-isolated, oxidized form of the enzyme resides in the active Ni_a_-S state (Ni^II^, S = 0),^13^,^16^–^18^ which is characterized by an open binding site between nickel and iron.^13^,^14^,^25^ In the current study, novel insights into this H_2_-accepting catalytic intermediate of [NiFe] hydrogenases are provided. Using ultrafast and 2D-IR spectroscopies, we shed light on key bond properties of the CO ligand, dynamic interactions between the diatomic ligands, and the complex interplay between the [NiFe] active site and its protein environment.

## Results and discussion

**Fig. 2:**
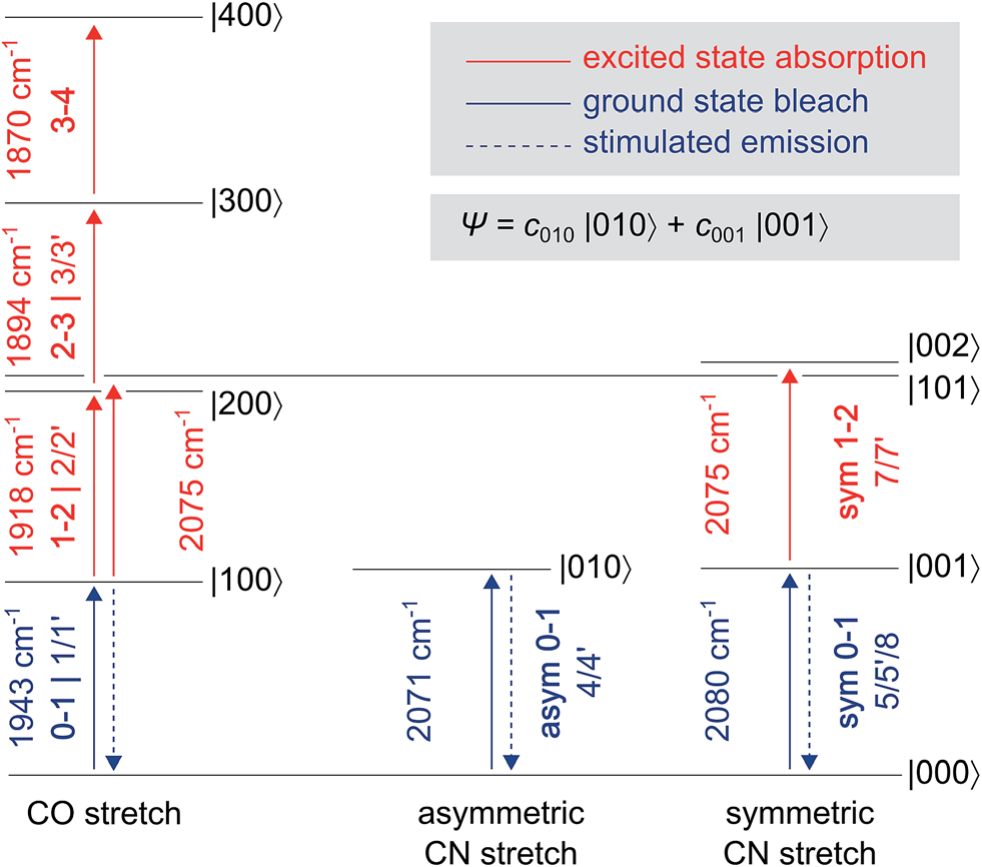
Eigenstates |*abc*〉 and transition energies (drawn to scale) of the CO/CN stretch vibrational manifold, as detected for oxidized and transition energies (drawn to scale) of the CO/CN stretch vibrational manifold, as detected for oxidized *Re*RH in the Ni_a_-S state. *a*, *b*, and *c* refer to the number of vibrational quanta in the CO stretch mode, the asymmetric CN stretch mode, and the symmetric CN stretch mode, respectively. As discussed in the main text, a common ground state |000 refer to the number of vibrational quanta in the CO stretch mode, the asymmetric CN stretch mode, and the symmetric CN stretch mode, respectively. As discussed in the main text, a common ground state |000〉 can be assumed. Transitions are labelled with the signal descriptors used in the pump–probe ( can be assumed. Transitions are labelled with the signal descriptors used in the pump–probe (Fig. 3) and 2D-IR (Fig. 6) spectra. Note that 2D-IR signal **6** is not included as it has contributions from several transitions, and the associated eigenstate energies are not precisely known (Fig. S4†). A non-stationary (coherent) state representing a linear combination of CN stretch excited states is listed separately.

**Fig. 3:**
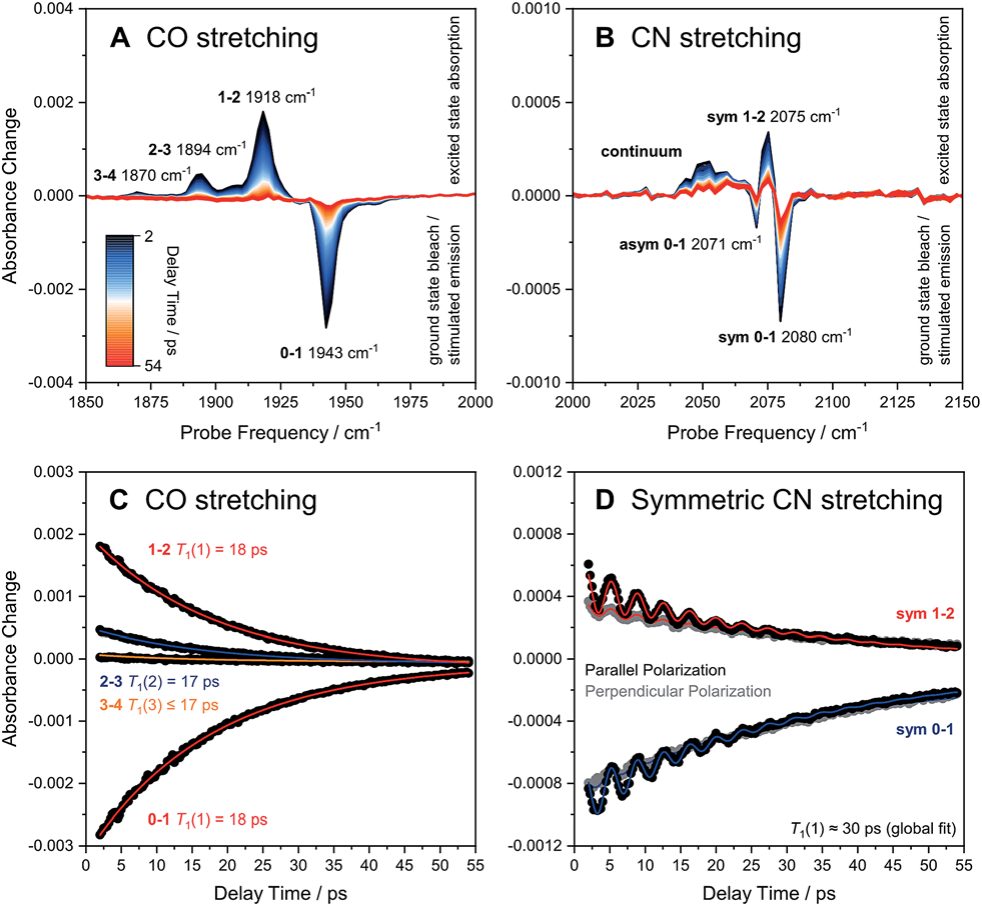
Broadband pump–probe spectra (A and B) and time evolution of selected signals (C and D) as obtained for oxidized *Re*RH in the Ni_a_-S state. Data referring to CO and CN stretch modes are plotted separately on the left (A and C) and right (B and D), respectively. Unless indicated otherwise, all data were acquired with a parallel pump–probe polarization geometry.‡ Transitions between vibrational eigenstates |*m*〉 and | and |*n*〉 are labelled as are labelled as **m–n**. For the CO stretch mode (C), vibrational lifetimes *T*_1_(*n*) of eigenstates |*n*〉 were obtained by fitting monoexponential decay curves (coloured lines) to experimental were obtained by fitting monoexponential decay curves (coloured lines) to experimental **m–n** time traces (black dots). For the symmetric CN stretch mode (D), *T*_1_(1) was determined by globally fitting the sum of a monoexponential decay curve and a monoexponentially damped sine function (coloured lines) to experimental **0–1** and **1–2** time traces obtained with both parallel (black dots) and perpendicular polarization (grey dots). sym, symmetric CN stretch mode; asym, asymmetric CN stretch mode.

The *Re*RH active site produces an IR absorption spectrum containing three peaks (Fig. 1, top) at 1943, 2071, and 2080 cm^–1^ that have been assigned to the fundamental transitions (ground to first excited state; *v* = **0–1**) of one CO and two CN stretching vibrations (Fig. 1, bottom; Fig. 2).^4^,^5^,^13^,^16^–^18^ IR pump–probe spectra are generated by successive interaction of the sample with two broadband (>300 cm^–1^) femtosecond-duration IR laser pulses. The pump pulse excites all molecular vibrations within the bandwidth of the laser, and the response of the sample to this perturbation is monitored by the probe pulse as a function of pump–probe delay time. IR pump–probe spectra are presented as difference spectra (Fig. 3, top) reflecting the change of probe light absorption by the sample following interaction with the pump pulse.‡
‡Pump–probe polarization geometries (parallel/perpendicular/magic-angle) refer to the angle between electric field vectors of the two pulses. Due to the huge size of native, double-dimeric *Re*RH (177.8 kDa),^17^ molecular rotation on the timescale of the IR pump–probe experiments is assumed to be negligible. Thus, analysis of vibrational lifetimes does not require spectra recorded under magic angle polarization *per se* (see SI3†).


In the region of CO stretching vibrations (1850–2000 cm^–1^), the IR pump–probe spectrum of oxidized *Re*RH contains multiple peaks (Fig. 3A). A negative feature at 1943 cm^–1^ is assigned to bleaching and stimulated emission of the *v* = **0–1** transition of the CO stretching vibrational mode (Fig. 1 and 2). This signal arises from population of the first excited state (*v* = **1**) by pump pulse excitation. The same process gives rise to enhanced absorption (positive peak) at 1918 cm^–1^ which is assigned to the *v* = **1–2** transition of the CO stretching vibration. Likewise, smaller positive features at 1894 and 1870 cm^–1^ are assigned to the *v* = **2–3** and *v* = **3–4** transitions, following population of the second and third vibrationally excited state, respectively (Fig. 2 and 3A).^26^,^27^

**Fig. 4:**
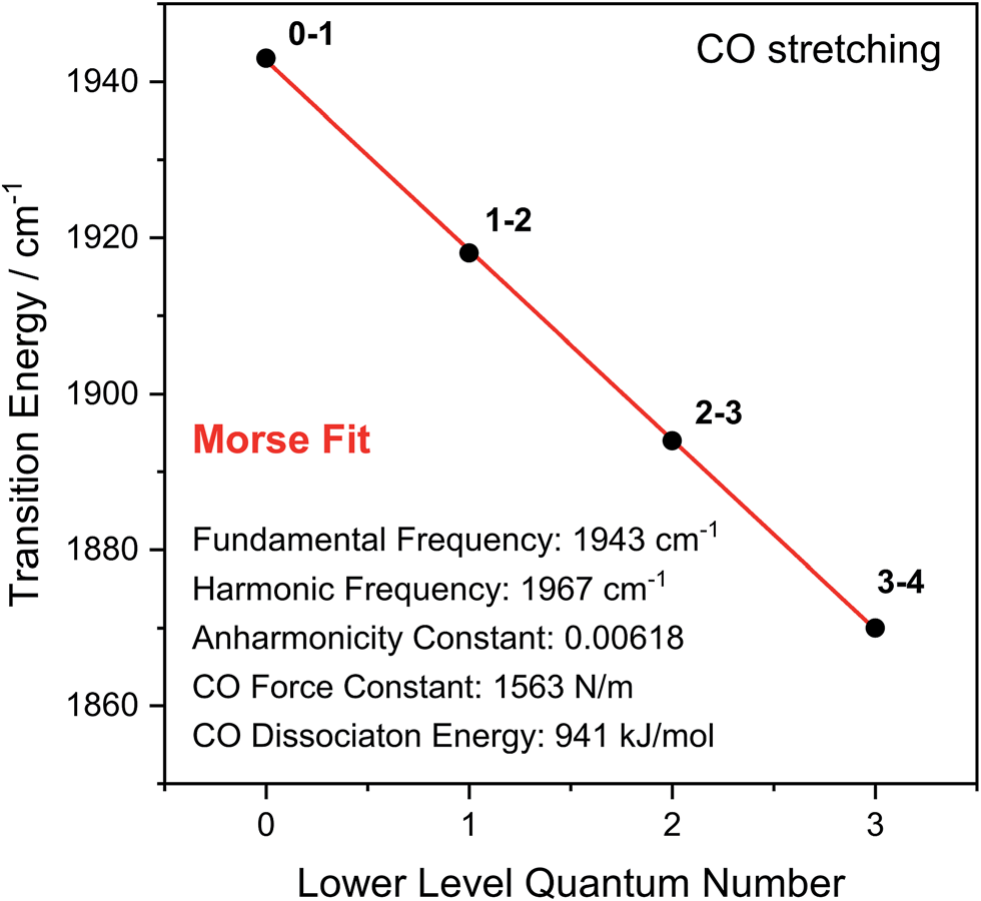
Transition energies observed for the CO stretch vibration of oxidized *Re*RH in the Ni_a_-S state. Indicated bond properties were determined by fitting a function representing energy level separations of a Morse potential (red line) to the experimental data (black dots). Transitions between vibrational eigenstates |*m*〉 and | and |*n*〉 are labelled as are labelled as **m–n**. For details, see SI2.†

Through observation of transitions from higher lying vibrational states (*v* > **1**), IR pump–probe spectroscopy allows experimental insights into the potential energy surface along the CO bond coordinate that go beyond IR absorption methods. Due to the constant spacing between transition energies (*i.e.* signals in the pump–probe spectrum), the metal-bound and protein-embedded CO ligand can be treated as an anharmonic diatomic molecule (see SI2†). Fitting a Morse potential^28^ to the experimentally observed transition energies, we obtain direct insights into fundamental properties of the CO ligand including bond strength and anharmonicity (Fig. 4). As a Lewis-amphoteric ligand,^29^,^30^ CO is capable of balancing the electron density at the active site, which is likely relevant for H_2_ binding and activation as well as the tuning of ground and transition state energies of species containing bridging hydrides. Thus, these insights into CO bonding promise to further our structural understanding of the [NiFe] active site and its rearrangement during catalytic turnover. In addition, these observables can be used as new structural markers to complement the conventional analysis of fundamental vibrational frequencies in hydrogenase research. Finally, the Morse fit also reveals the harmonic vibrational frequency, *i.e.* the quantity that is typically calculated in, *e.g.*, density functional theory (DFT) studies. Remarkably, this frequency is higher than the experimental transition energy by 24 cm^–1^. This significant effect has not been considered in any of the previously published theoretical studies on hydrogenases, which has far-reaching implications for the future implementation and interpretation of theoretical vibrational analyses in hydrogenase research.

IR pump–probe spectra obtained at higher frequencies (2000–2150 cm^–1^) reveal negative peaks that can be assigned to ground state bleaching and stimulated emission (**0–1**) of the asymmetric (2071 cm^–1^) and symmetric (2080 cm^–1^) CN stretching vibrational modes (Fig. 1 and 3B). While transient absorption from the first excited state (**1–2**) is clearly resolved for the symmetric stretching mode at 2075 cm^–1^ (Fig. 2 and 3B), the corresponding signal of the asymmetric mode is obscured by a broad continuous feature between *ca.* 2040 and 2070 cm^–1^. Due to strong overlap of the closely spaced and apparently less anharmonic CN stretch vibrations, these transitions will be discussed in more detail on the basis of 2D-IR spectra (*vide infra*).

The time-evolution of signals in the pump–probe spectra provide information on the lifetimes of vibrationally excited states (*T*_1_). These quantities reflect the rate of vibrational energy transfer away from the active site, thereby providing novel vibrational markers that yield insights into the dynamic active site environment. Apart from one known exception,^31^ CO and CN stretch frequencies are only sensitive towards isotope exchanges on the diatomic ligands themselves,^4^,^5^ due to the highly localized nature of these normal modes. In contrast, *T*_1_ may be highly sensitive towards substrate, protein, or solvent isotope exchanges,^32^–^35^ thereby allowing direct structural insight beyond the diatomic ligands. The intensities of both the **0–1** and **1–2** signals in the CO probe region of the spectra (Fig. 3A) are well modelled by mono-exponential decay curves (Fig. 3C), revealing an upper limit of 18 ps for the *v* = **1** state lifetime (Fig. 3C). This small value reflects fast intramolecular energy dissipation upon CO bond distortion, which could be relevant in case of fast catalytic steps that would be otherwise hampered by barrier recrossing. For a harmonic oscillator that is linearly coupled to a harmonic bath, vibrational lifetimes decrease linearly with the vibrational quantum number,^36^ as observed for a mononuclear homoleptic metal carbonyl compound in solution.^26^ In contrast, higher excited states of the CO stretch mode of *Re*RH decay at comparable rates, indicating considerable deviation from this relaxation model.

Notably, the CO vibrational lifetime of *Re*RH differs from most previously studied synthetic carbonyl and cyanido complexes.^26^,^27^,^33^–^35^,^37^–^41^ The observed value of 18 ps is significantly shorter than those typically observed for synthetic systems in non-interacting or hydrophobic solvents^39^,^41^ but longer than those of metallocarbonyls studied in water.^35^ Since energy dissipation from the deeply buried active site cannot proceed *via* collision with solvent molecules, vibrational relaxation likely involves anharmonic coupling^42^ of the CO stretch coordinate through σ or π bonding interactions with the Fe ion, as observed for comparably fast-relaxing carbonyl derivatives of heme proteins and related metalloporphyrins.^43^ In line with this proposal, DFT calculations on an [FeFe] hydrogenase model compound revealed pronounced anharmonic coupling between CO/CN stretch modes and low-frequency Fe(CO)(CN)_2_ metal–ligand vibrations that are observed experimentally for [Fe], [FeFe], and [NiFe] hydrogenases.^13^,^40^,^44^,^45^ It is thus tempting to propose that the short CO lifetime is due to through-bond energy redistribution towards the protein matrix, possibly involving the hydrogen-bonded CN^–^ ligands (*vide infra*) or the bridging cysteines. This conclusion highlights the role of the protein matrix in defining active site characteristics and explains the considerably longer relaxation times observed for non-proteic dithiolato-bridged carbonyl compounds in weakly interacting solvents.^39^,^41^

**Fig. 5:**
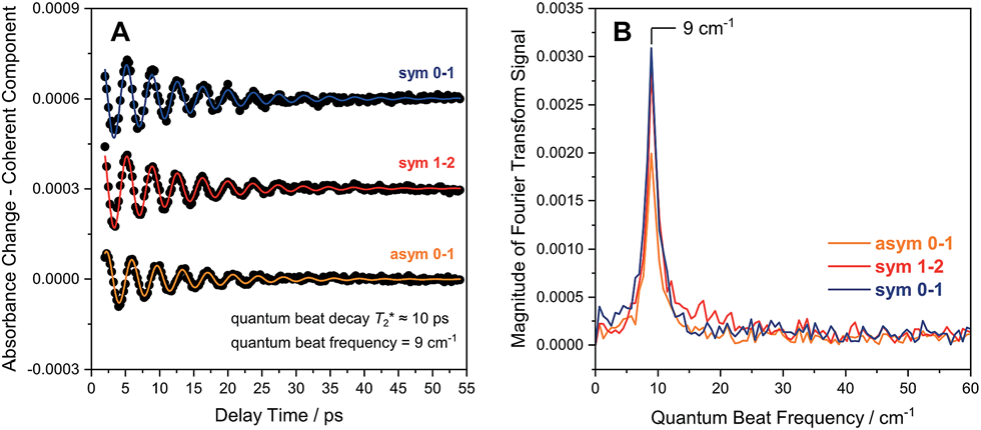
(A) Quantum beat patterns observed for CN stretch transitions of oxidized *Re*RH in the Ni_a_-S state. Data are shown stacked, and a monoexponential decay background has been subtracted from the raw data (Fig. 3D) to eliminate non-coherent contributions. Quantum beat frequencies and decay times were determined by fitting monoexponentially damped sine functions (coloured lines) to the experimental data (black dots). (B) Fourier transform of the time traces shown in (A). Data relating to transitions between vibrational eigenstates |*m*〉 and | and |*n*〉 are labelled as are labelled as **m–n**. Sym, symmetric CN stretch mode; asym, asymmetric CN stretch mode.

Due to smaller intensities, strongly overlapping signals of opposite sign, and kinetic complexity (*vide infra*), the extraction of vibrational lifetimes is more challenging for the two CN stretch modes (Fig. 3D). First-excited-state lifetimes of both vibrations are on the same order of magnitude but seem to be slightly longer than that of the CO stretch mode. Based on extensive analyses (see ESI: SI3†), we conclude that the apparent lifetime of the symmetric CN stretch mode is *ca.* 30 ps (Fig. 3D), and a similar value can be assumed for the asymmetric counterpart. The decay curves of all signals associated with the two CN stretch modes exhibit a pronounced oscillatory modulation, which is best observed after subtracting a mono-exponential background (Fig. 5A). This quantum beat pattern results from coherent excitation of the two CN stretch modes, which creates a superposition of their first excited states.^46^–^48^ The coherent superposition state decays faster than the individual excited states, indicating that the observed time constant of 10 ps can be associated with its pure dephasing time 
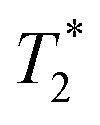
. As expected,^46^–^48^ the quantum beat frequency (9 cm^–1^) equals the difference between fundamental transition energies of the two involved modes (2080–2071 cm^–1^). Thus, analysis of quantum beats may allow unambiguous assignment of sets of signals to individual redox-structural states of the active site, even in complex (catalytic) mixtures. This is best achieved by Fourier analysis of the coherent decay curves, which yields well-defined frequency components for each pair of coherently excited vibrations (Fig. 5B). This finding is highly relevant, *e.g.*, for the detailed analysis of the CN stretching vibrations of [NiFe] hydrogenases, since these modes are valuable structural markers but typically hard to analyse due to weak intensity and strong overlap.^3^,^6^,^21^,^23^ Quantum beat analysis can circumvent this difficulty by revealing if (and to which degree) the splitting between the two CN stretch modes differs among constituents of a complex mixture. This may allow assignment of individual species *ad hoc* or provide physically grounded boundary conditions for otherwise ambiguous global band fit analyses of the IR spectra. The observation of quantum beats also implies a common vibrational ground state,^46^–^48^ revealing that the two CN stretch modes of [NiFe] hydrogenases are anharmonically coupled.§
§Notably, anharmonic coupling *between* symmetric and asymmetric stretch modes should not be confused with the well-known coupled stretching of the two CN bonds *within* both modes.^5^ While the latter aspect is well captured by harmonic vibrational analyses (see, *e.g.*, ref. 13, 19 and 21), the experimental observation of anharmonic coupling illustrates a partial breakdown of the harmonic approximation.


To obtain additional information on the interactions between the CO and CN stretching modes, 2D-IR spectra were obtained at a selection of waiting times *T*_w_ between pump and probe events. While these spectra were obtained using a Fourier transform (time domain) technique,^49^–^51^ their general features are best explained using pump–probe (frequency domain) terminology for consistency with earlier results.^10^,^52^ In contrast to broadband pump–probe spectroscopy, a 2D-IR spectrum provides frequency resolution of both pump and probe events, spreading the information along a second (pump) frequency axis.

**Fig. 6:**
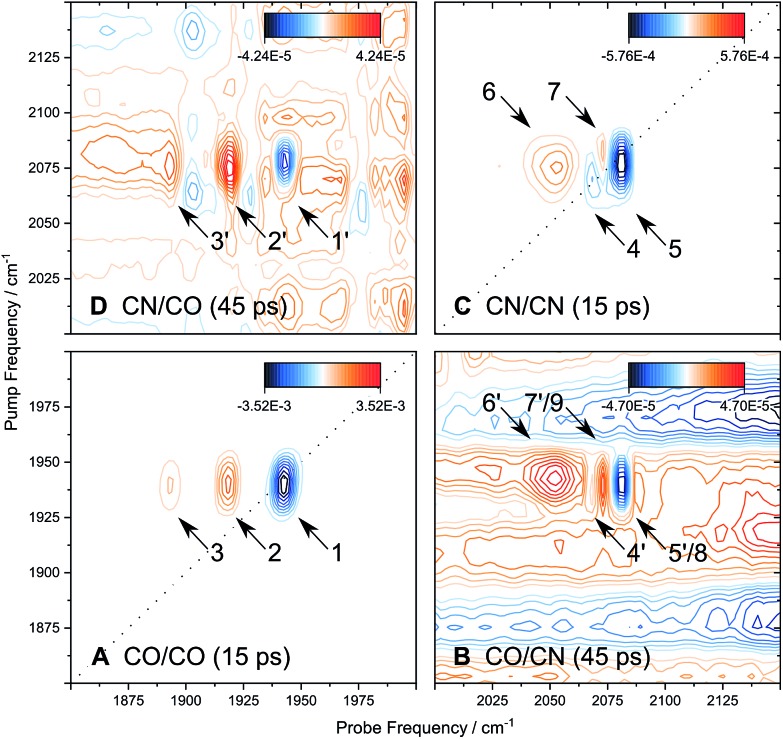
2D-IR spectrum of oxidized *Re*RH in the Ni_a_-S state, recorded with parallel polarization. Quadrants A to D reflect different pump and probe regimes, as indicated. Waiting times *T*_w_ are listed for each quadrant. Signals are numbered, and those reflecting vibrational energy transfer are marked with a prime. For details, see text.

**Fig. 7:**
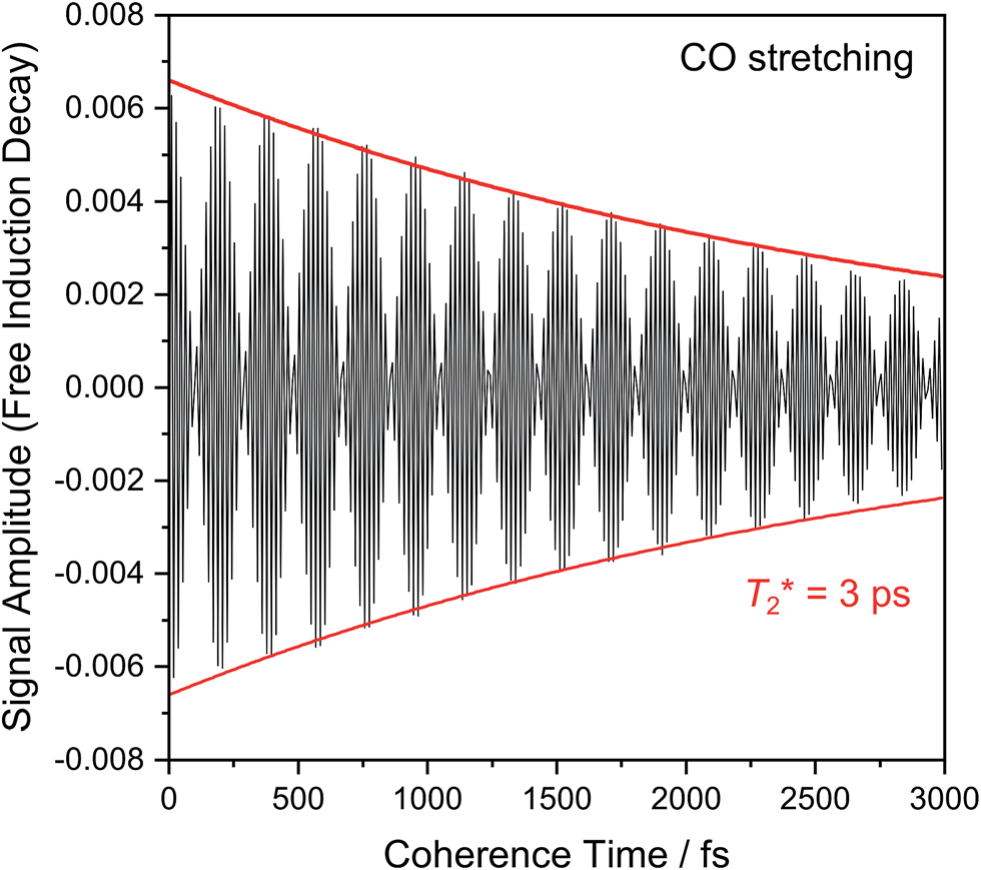
Evolution of 2D-IR signal intensity (black line) as a function of coherence time *τ*, modelled as a monoexponential decay curve (red lines). Data refer to the CO stretching mode (probe frequency: 1943 cm^–1^).

Fig. 6 shows the 2D-IR spectrum of oxidized *Re*RH in the Ni_a_-S state, split into four quadrants. Diagonal quadrant A exhibits signals resulting from pumping and probing the CO stretch mode, at *T*_w_ = 15 ps. The peaks present closely resemble those detected in the pump–probe spectrum at all waiting times (Fig. 3A). Specifically, peak **1** corresponds to **0–1** ground state bleaching and stimulated emission, while signals **2** and **3** can be assigned to the **1–2** and **2–3** transient absorption transitions, respectively (Fig. 2). A signal corresponding to the **3–4** transition is also present (Fig. S1†) but not visible using the contours shown in Fig. 6A. Due to higher frequency resolution along the probe axis, all signals are slightly ellipsoidal along the pump axis. No significant diagonal elongation of the peaks was observed though, and the diagonal peak shapes were not found to change with *T*_w_, indicating that little spectral diffusion is occurring.^10^,^12^ Inspection of the τ-dependence of the 2D-IR signal arising from the CO stretching mode indicates a 3 ps coherence decay for the **0–1** transition (Fig. 7). Within the homogeneous limit, this decay could be associated with the pure dephasing time 
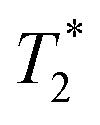
 since population relaxation is considerably slower (*T*_1_ = 18 ps, *vide supra*). This assignment, however, would imply a homogeneous linewidth of 3.5 cm^–1^,^12^,^53^ which is less than the experimental value of 7 cm^–1^. Likewise, the 10 ps pure dephasing time of the CN stretch modes (Fig. 5; *vide supra*) would correspond to a homogeneous width of 1 cm^–1^, while the experimental value is 6 cm^–1^. Based on these observations, we conclude that the experimental linewidths reflect moderate inhomogeneous broadening due to a mixture of structurally confined microstates. While fast (multi-scale) dynamics, as observed for a hydrogenase mimic,^54^ cannot be excluded, inhomogeneous distributions of ≤7 cm^–1^ imply that the underlying microstates convert slowly (≫10 ps),^12^ possibly indicating a multi-minima potential energy surface. Notably, narrow signals in vibrational spectra of hydrogenases have been previously ascribed to conformational constraints imposed by the protein,^44^,^55^,^56^ which is only valid within the inhomogeneous limit.^12^,^53^ Based on the high time resolution of the non-linear IR methods employed here, we can verify this implicit assumption, demonstrating that hydrogenase active sites are indeed geometrically tuned by the protein matrix. Notably, such a situation has been proposed to be catalytically relevant for efficient H_2_ binding to the central Ni_a_-S intermediate studied here.^13^,^57^

Signals in diagonal quadrant C of Fig. 6 reflect transitions that arise from the two CN stretch vibrations (*T*_w_ = 15 ps). Again, these signals resemble those in the pump–probe spectrum (Fig. 3B), but additional information can be extracted. Signals **4** and **5** can be assigned to the **0–1** transitions of the asymmetric and symmetric CN stretch mode, respectively (Fig. 2). The latter feature is elongated towards lower pump frequencies at all waiting times, indicating the presence of a cross peak related to anharmonic coupling between the two CN modes,^10^,^12^ in line with a shared vibrational ground state (*vide supra*).^46^–^48^ At long waiting times (*T*_w_ = 20 ps), peaks **4** and **5** extend towards higher and lower pump frequencies, respectively, in 2D-IR spectra recorded with perpendicular polarization (Fig. S3†). This observation reveals the presence of energy transfer between the two CN stretch modes,^10^,^12^ and the observed polarization dependence indicates an orthogonal arrangement of their transition dipole moments, as expected for symmetric and asymmetric modes arising from two CN oscillators. Signal **6** corresponds to the broad continuous feature observed in the pump–probe spectrum (Fig. 3B). Interestingly, this signal covers an extended range of pump frequencies as well, and the apparent band maximum shifts to higher probe frequencies as a function of *T*_w_ (Fig. S4†), indicating contributions from multiple excited state transitions. According to Fig. S4,† our observations are consistent with contributions from **2–3** and **3–4** transitions of the symmetric stretch mode and the **1–2** transition of the asymmetric stretch mode. In this respect, it should also be noted that the vibrational potentials of the CN stretching modes are likely different from that of the CO stretching vibration. Specifically, the Morse potential is clearly unsuited to describe asymmetric bond stretching, and transition energies may decrease more drastically with increasing quantum numbers than expected from this model.^27^ Furthermore, the anharmonicity of the symmetric stretch mode seems to be underestimated in the pump–probe spectrum, as indicated by strong overlap and partial cancelling of signals **4** and **7** in the 2D-IR spectrum (Fig. 6C). While additional effects cannot be categorically ruled out,^37^ these aspects explain why higher excited state transitions of the CN stretch modes give rise to an unexpectedly broad and unresolved feature rather than well-defined sharp peaks, as observed for CO stretching (Fig. 3A and 6A). Given the lack of a second frequency axis, all this information is inaccessible from the pump–probe data, highlighting the importance of 2D-IR spectroscopy for disentangling complex vibrational signatures of hydrogenases.

We next turn to the off-diagonal quadrants of the 2D-IR spectrum, B and D, which reflect interactions between CO and CN stretching modes. Specifically, quadrant B (D) reflects changes in CN (CO) stretch absorbance due to pumping the CO (CN) stretch modes. At long waiting times (*T*_w_ = 45 ps), quadrant D exhibits signals **1′**, **2′**, and **3′** that reproduce diagonal features **1**, **2**, and **3** (Fig. 6A). Being absent at early waiting times (data not shown), these off-diagonal features can be ascribed to vibrational energy transfer from the CN^–^ ligands towards the CO ligand,^10^,^12^ and the reverse process can be inferred from signals **4′**, **5′**, **6′**, and **7′** in quadrant B. This confirms that energy from the excited CO stretch mode may be (covalently) dissipated towards the CN^–^ ligands and *vice versa*. Interestingly, negative signals **8** and **9**, coinciding with **5′** and **7′**, can be observed from earliest waiting times on, indicating that there is anharmonic coupling between CO and (symmetric) CN stretch modes (Fig. S2†).^10^,^12^ Due to the larger transition dipole moment of the pumped CO stretch mode, signal intensities in quadrant B approach those of diagonal CN stretch signals **4**, **5**, **6**, and **7**. According to the projection slice theorem,^12^ these off-diagonal features may therefore contribute to the broadband pump–probe data in a nonnegligible manner, which explains the kinetic complexity observed for CN stretch signals in these spectra (*vide supra*). Considering that signals **4′**, **5′**, **6′**, and **7′** grow into the 2D-IR spectra as a function of *T*_w_, apparent vibrational lifetimes of the CN stretch modes – as determined from pump–probe spectra – likely represent upper limits. In total, pump–probe and 2D-IR data indicate that CO and CN stretch vibrations relax comparably fast within few tens of picoseconds, indicating that energy from both modes is efficiently dissipated *via* similar (covalent) pathways towards the protein matrix.

## Conclusions and outlook

In the current account, we have demonstrated how ultrafast and multi-dimensional IR techniques can be used to obtain detailed insights into the structure and dynamics of hydrogenases. Utilizing CO/CN stretch vibrations as ideal IR probes, these methods were shown to provide access to several unexplored observables that can be used as novel vibrational markers to study the active sites of these enzymes, as summarized in the following.

(1) Using an O_2_-tolerant [NiFe] hydrogenase as a model system, we have demonstrated that transitions between multiple excited vibrational states can be used to extract detailed information on CO bond properties that are directly linked to fundamental physical quantities and inaccessible by other experimental techniques (Fig. 4). This approach will assist in understanding how CO bonding controls catalytic H_2_ binding and cleavage,^29^,^30^ as exemplified for the Ni_a_-S state, the H_2_-accepting intermediate of [NiFe] hydrogenases. Notably, this analysis also revealed that the harmonic frequency is clearly higher than the experimental value. This supports the previous notion that relative rather than absolute frequencies should be analysed in theoretical studies on, *e.g.*, hydrogenases.^23^ Moreover, the experimental frequency should not be interpreted as a quantitative bond strength measure since the simple relation between this observable and the bond force constant is strictly valid within the harmonic approximation only (see SI2†). (2) Analysing the coherence decay of CO and CN stretch modes (Fig. 5 and 7), we have gained insights into the magnitude and time scales of equilibrium fluctuations, thereby revealing a structurally confined environment for both types of ligands. Given the high sensitivity of their stretching frequencies towards structural features of the entire [NiFe] centre,^3^,^6^,^21^,^23^ this finding shows that hydrogenase active sites are geometrically tuned by protein structural constraints, as claimed to be mandatory for H_2_ binding to Ni_a_-S.^13^,^57^ Similar analyses promise to yield detailed insights into the structural plasticity of native and artificial maturation intermediates, whose vibrational linewidths are typically much broader than those of mature hydrogenase active sites.^44^,^55^,^56^,^58^ (3) In principle, CN stretch frequencies are highly sensitive probes, *e.g.*, for structural changes affecting the equatorial plane of the Fe site in [NiFe] hydrogenases.^21^,^23^ In these enzymes, however, their assignment and interpretation is typically complicated due to overlap of closely spaced low-intensity signals from all probed intermediates in a (catalytic) mixture of states. We have shown that such challenges can be addressed by quantum beat analysis, which allows an unambiguous assignment of CN stretch modes (Fig. 5). This approach is further facilitated by cross peaks in the 2D-IR spectra, which clearly correlate CO and CN stretch signals corresponding to a single redox-structural state (Fig. 6). (4) These cross peaks also provide insights into the extensive interplay between individual vibrations, thereby helping to properly understand the CO/CN modes that have been utilized as structural markers for decades (Fig. 2). Specifically, all three stretch modes of [NiFe] hydrogenases are found to be anharmonically coupled, and energy can be quickly transferred both between them and towards the protein matrix, probably *via* a covalent route involving the bridging cysteine ligands. This finding is expected to be important for energy dissipation during fast, non-classical catalytic steps with notable CO/CN bond rearrangement, *e.g.*, H_2_ binding and cleavage. (5) In the future, 2D-IR cross peaks may also be utilized to gain direct structural insight beyond the diatomic ligands by analysing coupling and energy transfer with other marker vibrations that cannot be directly probed in IR absorption experiments. This approach may be further facilitated by the intrinsic sensitivity of many introduced observables to solvent, substrate, or protein isotope exchanges, which is expected to inform on, *e.g.*, hydrogen and oxygen adducts. This way, the structural analysis of CO/CN stretch modes can be rationalized and expanded without losing the intrinsic advantages of these highly localized reporter vibrations.

Despite the plethora of additional information available from nonlinear techniques, they retain the intrinsic advantages of IR spectroscopy, *i.e.* various sample forms can be probed in a non-invasive manner under physiologically relevant conditions and versatile experimental control. Moreover, sample requirements and acquisition times (see Experimental details) are able to compete with benchtop IR experiments, in contrast to other advanced (vibrational) spectroscopies. Remarkably, many of the outlined features were also observed for low-concentration samples that appeared almost IR-transparent in basic absorption experiments. This is due to the fact that the nonlinear nature of the 2D-IR signal amplifies narrow transitions with large extinction coefficients.^59^ Thus, nonlinear IR techniques are particularly suited for *in vivo* studies^20^,^22^,^23^,^60^ that are highly informative but often limited by the low hydrogenase content in living organisms. Such studies would particularly benefit from the sensitivity of 2D-IR spectroscopy towards solvent effects, intermolecular interactions, and other environmental factors.^10^

While demonstrated for [NiFe] hydrogenases, the outlined approaches can be easily adapted for the investigation of [FeFe] and [Fe] hydrogenases, CO/CN-bound maturation factors, and other enzymes or (bioinspired) catalysts that contain or interact with feasible IR-chromophores, *e.g.* nitrogenase, CO dehydrogenase, and NO reductase. Finally, we stress the exceptional time resolution of non-linear IR techniques, which exceeds all previous studies on hydrogenases by at least three orders of magnitude. Notably, this valuable trait is not limited to equilibrium studies but can also be utilized in nonequilibrium experiments. To this end, both techniques can be combined with an additional (visible) pulse, which allows triggering and probing entire reactions or individual elementary steps with ultra-high time resolution. This trait is of utmost importance for probing fast and so-far inaccessible catalytic events including H_2_ binding and cleavage, and even non-stationary structures along catalytic reaction coordinates come into reach.

## Experimental details

### Preparation of *Re*RH

*R. eutropha* HF574 (pGE#3888),^13^,^24^,^61^ overproducing double-tagged native (double-dimeric) *Re*RH, was cultivated heterotrophically in a mineral salt medium, as described previously.^13^,^62^ After cell harvest and disruption, *Re*RH was purified from the soluble extract according to established procedures.^13^,^17^,^24^ For spectroscopic studies, double-dimeric *Re*RH was ultrafiltrated (Amicon Ultra-100, Millipore) to a final concentration of *ca.* 0.6 mM (corresponding to a [NiFe] centre concentration of 1.2 mM) and stored at –80 °C until further usage.

### Ultrafast IR pump–probe and 2D-IR spectroscopy

Spectra were recorded in transmission mode using a gas-tight and temperature-controlled (*T* = 10 °C) small-volume sandwich cell (optical path length = 50 μm, *V* ≈ 8 μL) equipped with CaF_2_ windows. All data were acquired in (pseudo) pump probe geometry utilizing mid-IR pulses (centre frequency = 2000 cm^–1^; bandwidth > 300 cm^–1^; pulse duration = 50 fs; repetition rate = 10 kHz) from the ULTRA laser-system as described previously.^49^–^51^,^63^ Series of pump–probe spectra (accumulation time = 3 s) were recorded by scanning the pump–probe delay time from –10 to 54 ps (step size = 250 fs).

2D-IR spectra (accumulation time = 300 s) were obtained at several selected waiting times *T*_w_ between 250 fs and 50 ps. For each fixed *T*_w_, 2D-IR data were obtained in a time-domain fashion by scanning the coherence time *τ* between two pulse-shaper generated collinear pump pulses from 0 to 2997 fs (step size 9 fs) prior to overlap with the probe pulse and self-heterodyned detection of the collinearly emitted signal.^49^–^51^ The pump frequency axis was obtained by Fourier transformation of the time-domain signal with respect to *τ*, while the probe frequency axis of both 2D-IR and pump–probe spectra was obtained by signal dispersion in a spectrograph and detection *via* liquid-nitrogen cooled 128-element MCT (mercury-cadmium-telluride) detectors with a resolution of <2 cm^–1^. Four-frame phase cycling was employed in 2D-IR data acquisition to limit contributions from pump light scattered on the detector.^50^

## Conflicts of interest

There are no conflicts to declare.

## Supplementary Material

Supplementary information
