# Supplementary material for: Understanding the structure and dynamics of hydrogenases by ultrafast and two-dimensional infrared spectroscopy
Source: Chem Sci. 2019 Aug 5;10(39):8981–9. doi: 10.1039/c9sc02851j (PMC6857670; doi:10.1039/c9sc02851j)
Supplement: Supplementary file 1 [file SC-010-C9SC02851J-s001.pdf]

Electronic Supplementary Material (ESI) for Chemical Science.
This journal is © The Royal Society of Chemistry 2019

Understanding the Structure and Dynamics of Hydrogenases by Ultrafast and Two-Dimensional Infrared Spectroscopy

Marius Horch[1,2]*; Janna Schoknecht[2]; Solomon L. D. Wrathall[1]; Gregory M. Greetham[3]; Oliver Lenz[2]; Neil T. Hunt[1]

[1]Department of Chemistry and York Biomedical Research institute, University of York, Heslington, YO10 5DD, York, United Kingdom
[2]Institut für Chemie, Technische Universität Berlin, Straße des 17. Juni 135, D-10623, Berlin, Germany
[3]STFC Central Laser Facility, Research Complex at Harwell, Rutherford Appleton Laboratory, Harwell Science and Innovation Campus, Didcot, OX110PE, Oxford, United Kingdom

*marius.horch@york.ac.uk

Supplementary Information

Contents

SI1: Supplementary Figures (pp. S1–S4)

SI2: Description of the Morse Fit (pp. S5–S6)

SI3: Analysis of CN Stretch Vibrational Relaxation (pp. S7–S8)

References (p. S8)

SI1: Supplementary Figures

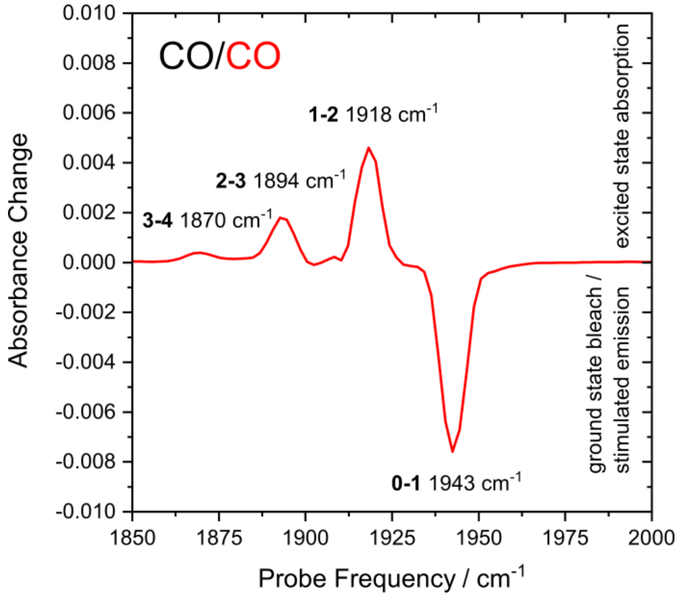

Figure S1: Horizontal slice (pump frequency = 1942 cm^{-1}) of the 2D-IR spectrum of oxidized *Re*RH in the Ni_a-S state, recorded at T_w = 250 fs. Data are shown for the CO stretch probe region, and transitions between vibrational eigenstates $|m\rangle$ and $|n\rangle$ of the CO stretch mode are labelled as **m-n**. Note that excited state absorptions are detected for vibrational levels $|1\rangle$ to $|3\rangle$, as observed in the broadband pump-probe spectra (cf. Fig. 3A).

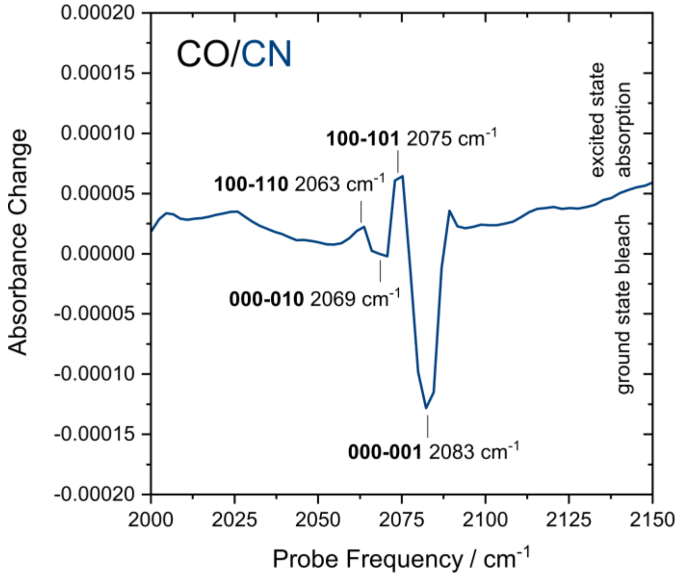

Figure S2: Horizontal slice (pump frequency = 1942 cm^{-1}) of the 2D-IR spectrum of oxidized *Re*RH in the Ni_a-S state, recorded at T_w = 250 fs. Data are shown for the CN stretch probe region, and transitions between vibrational eigenstates $|abc\rangle$ and $|xyz\rangle$ are labelled as **abc-xyz**. Here, a, b, and c (x, y, and z) refer to the number of vibrational quanta in the CO stretch mode, the asymmetric CN stretch mode, and the symmetric CN stretch mode, respectively.

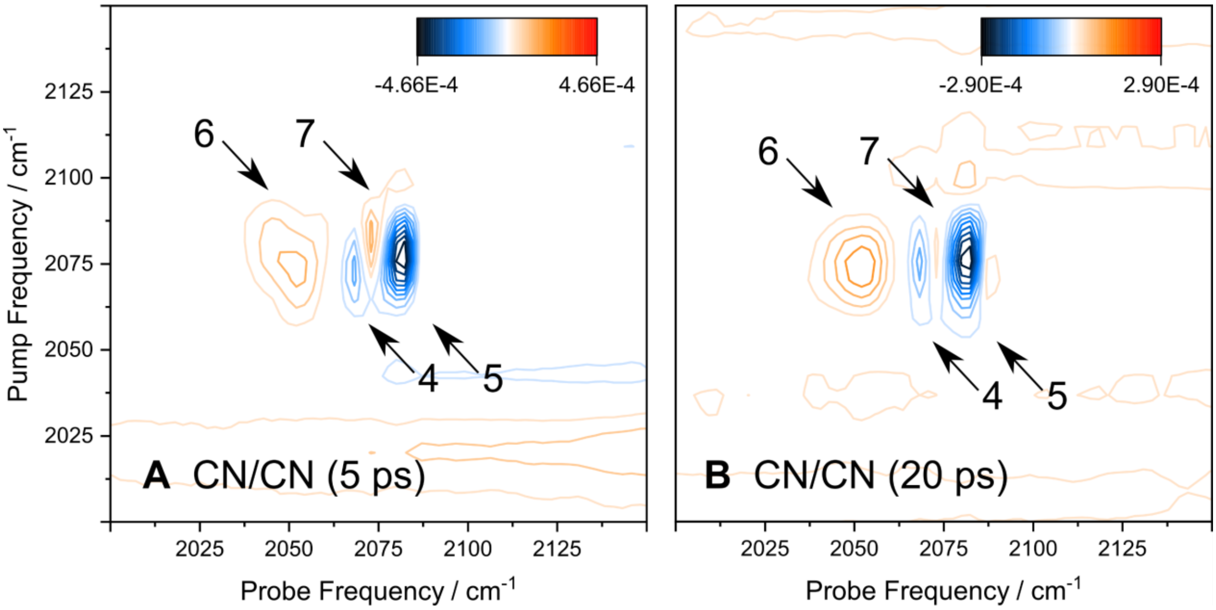

Fig. S3: 2D-IR spectra of oxidized *Re*RH in the Ni_a-S state, recorded with perpendicular polarization. Spectra show the CN stretch pump and probe regimes (cf. quadrant C of Fig. 6). Waiting times are indicated for each spectrum, and signals are numbered according to the nomenclature introduced in the manuscript.

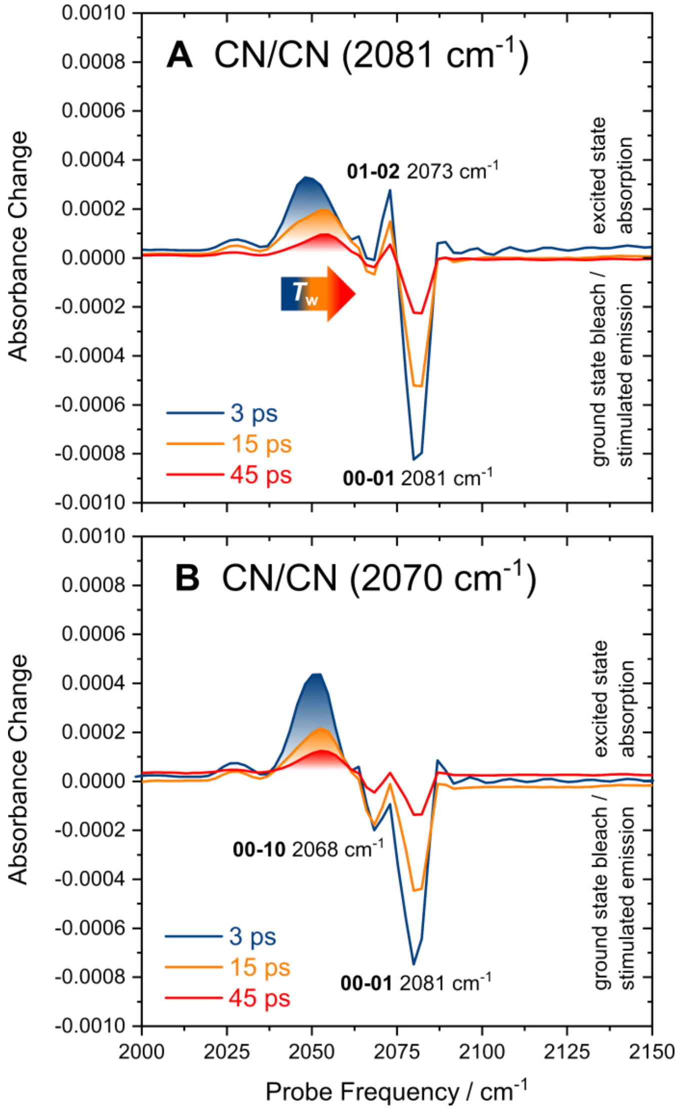

Figure S4: Horizontal slices of the 2D-IR spectrum of oxidized *Re*RH in the Ni_a-S state, obtained at the indicated waiting times. Data are shown for the CN stretch probe region and two indicated pump frequencies close to the absorption maxima of the symmetric (A) and asymmetric (B) CN stretching modes. At longer waiting times T_w, the apparent maximum of the broad continuum feature shifts towards higher frequencies in A (pump frequency = 2081 cm^{-1}). This can be explained by the faster relaxation of higher excited vibrational states, and the observed trend is consistent with contributions from at least two peaks. We tentatively assign these features to the **2-3** and **3-4** transitions of the symmetric CN stretching mode, the lowest otherwise unobserved transitions of this mode. In contrast, the band maximum in B (pump frequency = 2070 cm^{-1}) is virtually independent from T_w, which indicates dominant contributions from a single peak, most likely reflecting the otherwise unobserved **1-2** transition of the asymmetric CN stretching mode. Due to broadening along the pump axis, intense signals from the symmetric CN stretching mode also contribute at this lower pump frequency, which explains the high intensity and slight shift of the broad continuum feature in B. Transitions between vibrational eigenstates $|ab\rangle$ and $|xy\rangle$ are labelled as **ab-xy**, where a and b (x and y) refer to the number of vibrational quanta in the asymmetric and symmetric CN stretch mode, respectively.

SI2: Description of the Morse Fit

The empiric Morse potential [1] describes the potential energy V of an anharmonic diatomic molecule as a function of the bond length r relative to its equilibrium value r_0. A typical form of the potential is given by

$$V = D\left(1 - e^{-a(r-r_0)}\right)^2 \tag{1}$$

where D is the depth of the potential and a is related to the force constant k by

$$a = \sqrt{\frac{k}{2D}} \tag{2}$$

Approximate solutions for the vibrational eigenstate energies of a Morse oscillator are given by

$$E_n = h\nu_0\left(n + \frac{1}{2}\right) - \frac{\left[h\nu_0\left(n + \frac{1}{2}\right)\right]^2}{4D} \tag{3}$$

Here, h is the Planck constant, n is the vibrational quantum number, and ν_0 is the harmonic vibrational frequency, which is related to k and the reduced mass μ of a diatomic molecule by

$$\nu_0 = \frac{1}{2\pi}\sqrt{\frac{k}{\mu}} \tag{4}$$

Transition energies $\Delta E_{n+1\leftarrow n}$ between neighbouring vibrational levels are given by

$$\Delta E_{n+1\leftarrow n} = E_{n+1} - E_n = h\nu_0 - \frac{(n+1)(h\nu_0)^2}{2D} \tag{5}$$

Fitting this expression to a set of experimental transition energies, ν_0 and D can be directly obtained. In addition, k can be calculated using equation 4, and the dissociation energy D_0 is obtained by subtracting the ground state (zero-point) vibrational energy

$$E_0 = \frac{1}{2}h\nu_0 - \frac{(h\nu_0)^2}{16D} \tag{6}$$

from D. k and D_0 are bond strength descriptors that reflect a diatomic molecule's resistance towards (infinitesimal) displacement from r_0 and bond breakage, respectively. Notably, it is important to calculate k from the real harmonic frequency, as determined from equation 5. Plugging the experimental ground state absorption frequency into equation 4 instead would be highly misleading since k scales quadratically with ν_0, and the bond anharmonicity may vary among different redox-structural states of the hydrogenase active site.

Using equation 5, we can also calculate the spacing between two transitions (spectroscopic lines) of a Morse oscillator as

$$\Delta\Delta E = E_{n+2} - E_{n+1} - (E_{n+1} - E_n) = -\frac{(h\nu_0)^2}{2D} \tag{7}$$

This expression is noteworthy for two reasons: (1) For an ideal Morse oscillator, the spacing between *each* set of neighbouring transitions is a (quantum-number independent) non-zero constant, and the outlined Morse fit is only valid if the experimental spectroscopic lines reproduce this behaviour. This is the case for the analysed CO stretch mode of oxidized *Re*RH. (2) For the ground state absorption energy, we obtain

$$\Delta E_{1\leftarrow 0} = E_1 - E_0 = h\nu_0 - \frac{(h\nu_0)^2}{2D} = h\nu_0 + \Delta\Delta E \tag{8}$$

In other words, for a Morse oscillator, the harmonic vibrational frequency can also be obtained by simply adding the (unsigned) spacing between spectroscopic lines to the experimental ground state absorption frequency. This behaviour is, again, well reproduced by the CO stretch mode of oxidized *Re*RH, justifying the Morse fit analysis.

An alternative, empiric expression for the vibrational energy levels of an anharmonic diatomic molecule is given by

$$E_n = h\nu_0\left(n + \frac{1}{2}\right) - h\nu_0\chi\left(n + \frac{1}{2}\right)^2 \tag{9}$$

Combining equations 3 and 9, we obtain an analytical expression for the dimensionless (first order) anharmonicity constant

$$\chi = \frac{h\nu_0}{4D}$$

Note that many (historic) texts report $\nu_0\chi$ instead, typically in units of cm^{-1}.

SI3: Analysis of CN Stretch Vibrational Relaxation

In case of the CN stretch modes, quantitative analysis of vibrational relaxation is complicated by the presence of several overlapping narrow bands of opposite sign and the contribution of pronounced off-diagonal signals to the pump-probe data (Fig. 6). In the following, the global fit described in the manuscript will be motivated and discussed together with alternative strategies for data analysis.

In principle, excited state decay kinetics may be determined from the time evolution of pump-probe data either by evaluation of signal intensities at discrete frequency points (typically band maxima) or by band fit analysis yielding integral signal intensities. In the current case, the latter approach turned out to be infeasible due to overlapping signals of opposite sign that cancel at their facing edges. In an unconstrained band fit, this situation results in underestimated linewidths, distorted band shapes, and displaced band maxima. In principle, these problems can be circumvented by constraining, e.g., linewidths and/or band maxima to values determined from IR absorption spectra. This procedure, however, introduces considerable bias to the analysis, and neither linewidths nor band maxima are known for signals reflecting excited state absorption. Moreover, linewidths and band shapes turned to out to vary as a function of pump-probe delay time, due to the presence of pronounced quantum beat patterns (Fig. 3D; Fig. 5). Thus, all approaches described in the following are based on the analysis of signal intensities determined from individual frequency points (corresponding to band maxima unless stated otherwise).

Due to the large size of native, double-dimeric *Re*RH (177.8 kDa),[2] molecular rotation is negligible on the timescale of the experiments. Thus, accurate vibrational lifetimes should be extractable from measurements performed with either parallel or perpendicular polarization. Moreover, vibrational lifetimes determined from signals reflecting **0-1** and **1-2** transitions should be identical. Due to the difficulties mentioned above, however, nonidentical lifetimes were obtained for these scenarios from mono- and biexponential fits (including a monoexponentially damped sine term to account for the quantum beat pattern).[a] In case of the symmetric stretch mode, this problem can be addressed by setting physically reasonable boundary conditions. Specifically, data can be analysed by a global fit sharing decay constants and quantum beat frequencies between all four analysed time traces, i.e. **0-1** and **1-2** signal decay curves from spectra recorded with parallel and perpendicular polarization (Fig 3D). This approach yields apparent lifetimes of ca. 30 and 45 ps, respectively, for mono- and biexponential fits including monoexponentially damped sine terms. Due to kinetic complexity in the CN probe region,[b] biexponential fitting is more accurate; however, sharing of both decay constants between all four datasets is physically questionable but, unfortunately, necessary to obtain a mathematically stable solution. Thus, monoexponential analysis may be more reliable, indicating that the lifetime of the first excited state of the symmetric CN stretch mode is likely 30 ps or shorter (see manuscript). This conclusion is also supported by analysis of an isolated point at the high-frequency edge of the **0-1** signal of this mode (Fig. 3D). In the current case, intensities at this point are probably more reliable than those at band maxima as they are least affected by signal overlap and possible baseline drifts across the broad cluster of low-intensity bands in the CN stretch probe region (Fig.

[a] Analysing magic-angle data instead did not solve this problem, and results were comparable to those obtained from parallel and perpendicular data.

[b] Kinetic complexity results from the congestion of diagonal and off-diagonal signals in the CN stretch probe region of the broadband pump-probe spectra (see manuscript). Biexponential analysis could, in principle, solve this problem, but fits turned out to be mathematically underdetermined if parameters were not constrained or shared between datasets.

3B). Consistently, fitting a monoexponential decay curve to signal intensities at this frequency point yields a vibrational lifetime of ca. 30 ps as well. Notably, this finding is obtained without sharing fit parameters between datasets and independent from the chosen polarization (parallel, perpendicular, or magic angle) as well as the inclusion of a monoexponentially damped sine term accounting for the coherent part of the decay curves.

For the asymmetric CN stretch mode, neither of the two approaches is applicable since the **0-1** signal sits at the centre of the broad band cluster, and no well-defined **1-2** signal can be detected (Fig. 3B). An accurate evaluation of vibrational relaxation is furthermore complicated by the intrinsically lower signal intensity of the asymmetric CN stretch mode (Fig. 3B). However, its vibrational lifetime is likely similar to that of the symmetric CN stretch mode for the following reasons: (1) Both modes have contributions from the same internal (CN bond stretch) coordinates, and, thus, vibrational energy is likely dissipated *via* a similar route. (2) All analyses indicate vibrational relaxation of both CN stretch modes within tens of picoseconds. While the vibrational lifetime of the asymmetric stretch mode is predicted to be somewhat longer by the unconstrained fits, this finding is unlikely to be reliable if the intrinsic difficulties of this type of analysis are considered (*vide supra*). (3) Sharing decay constants and quantum beat frequencies between parallel and perpendicular datasets, a biexponential fit with a monoexponentially damped sine term yields comparable lifetimes for both modes (ca. 45 ps). For the quantitative determination of vibrational lifetimes, this approach is clearly inferior to those outlined above; however, it represents the most accurate analysis applicable to both modes, thereby allowing an estimate of their relative vibrational lifetimes.[c]

References

1 P. M. Morse, *Phys. Rev.*, 1929, **34**, 57.

2 M. Bernhard, T. Buhrke, B. Bleijlevens, A. L. de Lacey, V. M. Fernandez, S. P. J. Albracht and B. Friedrich, *J. Biol. Chem.*, 2001, **276**, 15592.

[c] Using this approach, comparable lifetimes are obtained from **0-1** and **1-2** signals of the symmetric CN stretch mode. This is not the case if a monoexponential fit is used instead, indicating that biexponential analysis is more favourable if fit parameters cannot be shared between **0-1** and **1-2** datasets (as is the case for the asymmetric CN stretch mode).
